# Dual block HER2 assessment increased HER2 immunohistochemistry positive rate in resected specimens of gastric cancer: a prospective multicenter clinical trial from China

**DOI:** 10.1186/s13000-022-01230-7

**Published:** 2022-06-28

**Authors:** Chen Xu, Miaomiao Sun, Mei Jin, Zengshan Li, Rong Qin, Guoping Ren, Wenyong Sun, Lirong Chen, Lijuan Luan, Yalan Liu, Dongxian Jiang, Lingli Chen, Rongkui Luo, Yingyong Hou

**Affiliations:** 1 Department of Pathology, Zhongshan Hospital, Fudan University, Shanghai, China; 2 Department of Pathology, Henan Cancer Hospital, Zhenghou, Henan China; 3 Department of Pathology, Sir Run Run Shaw Hospital, Zhejiang University, Hangzhou, Zhejiang China; 4 Department of Pathology, Xijing Hospital, Air Force Medical University, Xi’an, Shaanxi China; 5 Department of Pathology, The First Affiliated Hospital of Anhui Medical University, Anhui Hefei, China; 6 Department of Pathology, The First Affiliated Hospital of Zhejiang University, Hangzhou, Zhejiang China; 7 Department of Pathology, Zhejiang Cancer Hospital, Hangzhou, Zhejiang China; 8 Department of Pathology, The Second Affiliated Hospital of Zhejiang University, Hangzhou, Zhejiang China

**Keywords:** HER2, Gastric cancer, Immunohistochemistry, Multi-institutional study, Clinical trial

## Abstract

**Background:**

Former single center studies indicated that HER2 assessment with two primary tumor blocks (dual block HER2 assessment) could be an efficient and practical approach to overcome the adverse impact of heterogeneity and acquire a HER2 positive rate in gastric cancer (GC). This multicenter prospective clinical trial (NCT 02843412) was launched to verify its value and generality.

**Methods:**

A total of 3806 participants with primary GCs have been enrolled from 8 hospitals in China. Two primary tumor blocks were selected and recorded as block 1 and block 2 after histological evaluation. An HER2 (4B5) rabbit monoclonal antibody was used for the immunohistochemistry (IHC) analysis.

**Results:**

In total patients, HER2 IHC positive (3+) rate with dual block assessment (9.4%) was higher than that with single block assessment (block 1: 7.8%, block 2: 7.8%) (*P* < 0.001). Compared with single-block assessment, dual-block assessment increased the positive rate by approximate 20%. Similarly, HER2 equivocal (2+) rate was increased in dual block assessment (25.8%), which was higher than that in single block assessment (block 1: 20.3%, block 2: 20.9%) (*P* < 0.001). Conversely, dual block assessment demonstrated a lower HER2 negative (0/1+) rate (64.8%) than single block assessment (block1: 71.9%, block 2: 71.3%) (*P* < 0.001). These findings were also confirmed in individual hospitals.

**Conclusions:**

Dual block HER2 assessment effectively increased HER2 IHC positive rate in resected specimens of GC. We recommended dual block HER2 assessment be promoted in routine clinical practice in GC.

**Trial registration:**

ClinicalTrials.gov, NCT 02843412. Registered 1 July 2016 - Retrospectively registered.

**Supplementary Information:**

The online version contains supplementary material available at 10.1186/s13000-022-01230-7.

## Background

Gastric cancer remains a major health concern globally despite of a decline in the incidence and mortality rates recent years [1]. According to the GLOBOCAN 2018 data, it is the fifth most common malignancy worldwide and the third lethal cancer, with an estimated 783,000 deaths in 2018 [2]. Eastern Asia is a high-incidence geographical area, and in China, it is the second and the third malignancy for men and women respectively, and the second cause of cancer related death in both genders [3].

Prognosis of GC is generally dismal, mainly due to the biological behavior of the tumor and difficulty to identify early stage patients [4, 5]. For advanced/metastatic tumors, the 5-year survival rate varied from 5 to 20% with an overall survival (OS) of approximately 10 months after conventional chemotherapy [6, 7]. Efforts have been made to explore more effective regimens for GC [6]. In 2010, the ToGA trial proved that chemotherapy plus the molecular targeted regent trastuzumab significantly improved the OS of human epidermal growth receptor 2 (HER2) positive GC patients [8]. Since then, HER2 has been serving as an important predictive biomarker for the selecting eligible candidates for the targeted treatment.

The characteristics of HER2 have been studied thoroughly in GC recent years. Various approaches have been developed for HER2 test including immunohistochemistry (IHC), fluorescence in situ hybridization (FISH) and silver in situ hybridization (SISH) [9, 10]. Among them, IHC should be initial testing option and could identify most of the positive cases alone [11, 12]. One prominent feature of HER2 is that the heterogeneity is far more common in GC than in breast cancer (BC) [13–15]. The heterogeneity was estimated to be from 30% to up to 79.3% of HER2 positive cases [16, 17]. It adversely affects the accuracy of HER2 assessment and is the main reason to get the false negative results [18]. Therefore, evaluation of HER2 status can be regarded challenging in GC [19, 20].

To cope with the heterogeneity and get reliable HER2 results, we were the first to propose the idea of dual block HER2 assessment (using 2 primary tumor-containing blocks in the IHC staining of HER2) in resected specimens of GC [21]. We further proved that dual block assessment is an effective and efficient way to increase HER2 positive rate [22]. Nevertheless, all these former studies were single institutional studies and the conclusions as well as the generalizability are still to be elucidated in larger multi-institutional studies. Therefore, to further confirm the validity of dual block assessment in GC, we launched this multi-institutional prospective clinical trial.

## Methods and materials

### Study design

This was a multicenter prospective clinical trial (NCT 02843412) undertaken in 8 centers in China. The study was approved by the ethics board at the Zhongshan Hospital, Fudan University, Shanghai, China (B2015-055R). The study started on August 3, 2016 and ended on July 31, 2017. Resected specimens of histological confirmed gastric adenocarcinoma and adenocarcinoma of oesophagogastric junction were eligible for inclusion. After pathological evaluation, two tumor containing paraffin blocks from the primary site were selected for IHC staining of HER2.

Based on study design, cases that were diagnosed as adenocarcinoma were included. Major exclusion criteria included special subtypes (adenosquamous carcinoma, squamous carcinoma, hepatoid adenocarcinoma, and carcinoma with lymphoid stroma, and neuroendocrine tumors), having received neoadjuvant therapy prior to surgery, the presence of multiple tumors and/or recurrent tumors, and small tumor amount that confined to only one tumor block.

Main clinicopathologic parameters including patient age, gender, tumor location, Lauren classification, tumor differentiation, pTNM stage (according to the eighth edition of the Union for International Cancer Control (UICC) guidelines) were also collected.

### Specimen handling and histological evaluation

The resected specimens were fixed in 10% buffered formalin within 30 minutes after excision. The specimens were then processed with routine procedures after fixation for 24 hours. After fixation, the specimens were examined and handled based on the procedures that recommended in the *Rosai and Ackerman’s Surgical Pathology (10th Edithion)*. Haematoxylin and eosin (HE) staining was performed following the routine protocols in each center.

Histological evaluation was completed in each hospital to select eligible blocks for HER2 analysis. The HE sections were reviewed by two experienced gastrointestinal pathologists. Two primary tumor-containing blocks were selected for further HER2 assessment. Blocks were given priority in the selection if they contained an intestinal type tumor component (based on Lauren classification), demonstrated the lowest grade and were rich in tumor cells.

### IHC staining

In each center, the HER2 (4B5) rabbit monoclonal antibody (Ventana Medical Systems, Inc., Tucson, AZ, USA) was used for IHC staining of HER2. The staining was performed with iView DAB Detection Kits (Ventana, Tucson, AZ, USA) on BenchMark XT automated stainers (Ventana Medical Systems, Inc., Tucson, AZ, USA). To get reliable HER2 results, all centers followed the established staining procedures [22, 23]. Briefly, the tissue sections were firstly deparaffinized with EZ Prep (Ventana, Tucson, AZ) at 75 °C. Next, the sections were pretreated for antigen retrieval at 95 °C in Cell Conditioning 1 (Ventana, Tucson, AZ) using “standard cell conditioning”. Then, the sections were incubated with HER2 (4B5) primary antibody for 24 minutes at 37 °C after the endogenous peroxidase inactivation by hydrogen peroxide for 4 minutes. After primary antibody incubation, the sections were blocked using Endogenous Biotin Blocking Kit (Ventana, Tucson, AZ) and then incubated with a biotinylated secondary antibody for 8 minutes. A streptavidin–horseradish peroxidase conjugate was next added to the sections for 8 minutes at 37 °C. Finally, the slides undertook counterstain with Hematoxylin II (Ventana, Tucson, AZ) for 8 minutes and Bluing Reagent (Ventana, Tucson, AZ) for 8 minutes. Normal immunoglobulin G from the same species of primary antibody diluted to matching concentration of the primary antibody was used as the negative control. In each tumor slide, small pieces of GC tissue with HER2 IHC scoring 3+ and 0 were used as positive and negative controls.

### HER2 evaluation

All the slides were first reviewed by pathologists from each center and then they were re-reviewed by pathologists from Zhongshan Hospital, Fudan University. Each section was evaluated by two independent observers. A discussion panel including 3 observers was introduced for discrepant cases. HER2 status of both sections was evaluated separately for each case. HER2 results were recorded as block 1 and block 2 based on the order of the serial number generated in pathological sampling. The highest score was recorded as the final score of the case when discrepancies were found between the two blocks.

HER2 IHC status was evaluated following the previously established criteria for resected specimens of GC [14, 24, 25]. Briefly, cases with no staining or less than 10% tumor cell positive staining were scored 0; faintly or barely perceptible staining on ≥10% of tumor cell membrane and only in part of the membrane was assigned a score of 1+; weak to moderate complete, basolateral, or lateral membranous reactivity in ≥10% of tumor cells was considered to be 2+; and strong complete, basolateral, or lateral membranous reactivity in ≥10% of tumor cells was recorded as 3+. HER2 3+ was considered IHC-positive, 2+ was considered HER2-equivocal, and 0/1+ was considered HER2-negative.

### Statistics

χ2 tests were used for the univariate analyses. Cross-tabulations with qualitative variables between the two cohorts were analyzed with Pearson’s χ2 test. The McNemar test was used to compare single-block assessment and dual-block assessment. A *P* value < 0.05 was defined as statistically significant. No adjustments were made. The statistical package SPSS version 22.0 (SPSS, Inc., an IBM Company, Chicago, IL, USA) was used for all analyses.

## Results

### Patient characteristics

A total of 3806 cases were enrolled in the trial from 8 hospitals including Zhongshan Hospital, Fudan University (1389 cases); Henan Cancer Hospital (1044 cases); Sir Run Run Shaw Hospital, Zhejiang University (397 cases); Xijing Hospital, Fourth Military Medical University (336 cases); The First Affiliated Hospital of Anhui Medical University (237 cases); The First Affiliated Hospital of Zhejiang University (172 cases); Zhejiang Cancer Hospital (136 cases); The second affiliated Hospital of Zhejiang University (95 cases). The mean and median age was 61.6 and 63.0 years old in total patients. There were 2820 male and 986 female patients, respectively (male to female ratio 2.86:1). Other main clinicopathological parameters of total patients and each hospital were shown in Table 1 and Supplementary Table 1.

**Table 1:** Patient characteristics of total patients

	Total
Total, n (%)	3806 (100)
Gender, n (%)	
Male	2820 (74.1)
Female	986 (25.9)
Differentiation, n (%)	
Well	112 (2.9)
Moderate	751 (19.7)
Poorly	2942 (77.3)
Lauren, n (%)	
Intestinal	1350 (35.5)
Diffuse	1197 (31.5)
Mixed	1189 (31.2)
Indeterminate	70 (1.8)
Location, n (%)	
OGJ	186 (4.9)
U	1262 (33.2)
M	544 (14.3)
L	1802 (47.3)
Others	12 (0.3)
Stage, n (%)	
IA	573 (15.1)
IB	366 (9.6)
IIA	390 (10.2)
IIB	596 (15.7)
IIIA	788 (20.7)
IIIB	590 (15.5)
IIIC	497 (13.1)
IV	6 (0.2)
HER2, n (%)	
3+	358 (9.4)
2+	982 (25.8)
1+	1091 (28.7)
0	1375 (36.1)

*Abbreviations:* Intestinal: Intestinal type, *Diffuse* Diffuse type, *Mixed* Mixed type, *Indeterminate* Indeterminate type, *OGJ* Oesophagogastric junction, *U* The upper third of the stomach, *M* The middle third of the stomach, *L* The lower third of the stomach

### Basic characteristics of HER2 status

**Fig. 1:**
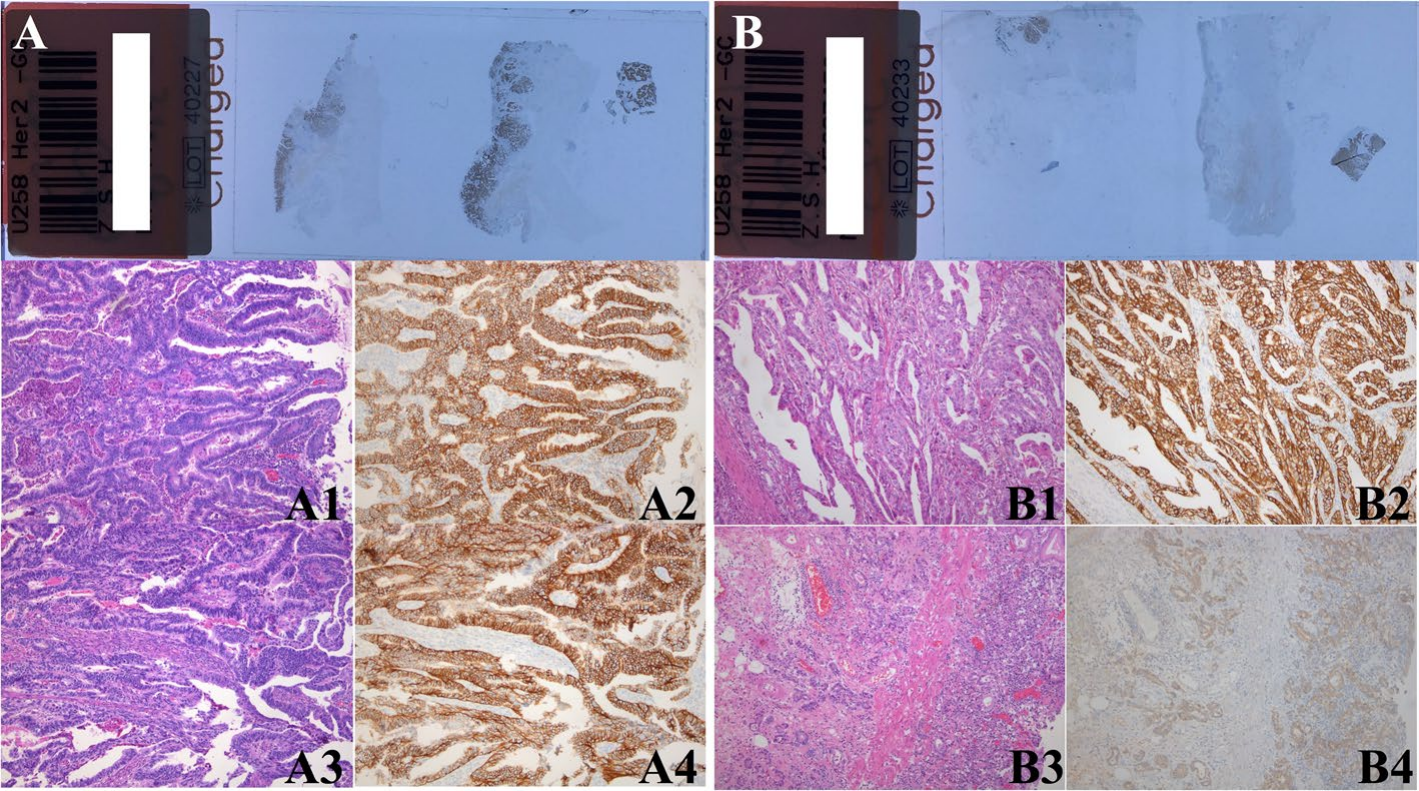
Examples of cases with concordant or discordant HER2 results. A. A concordant case. Block1 (A1, A2): 3+; Block2 (A3, A4): 3+. B. A discordant case. Block 1 (B1, B2): 3+; Block 2 (B3, B4): 1 +

All the patients undertook HER2 dual block assessment (Fig. 1). In total patients, there were 357 cases scoring HER2 3+ (9.4%), 982 cases scoring 2+ (25.8%), 1091 cases scoring 1+ (28.7%), and 1376 cases scoring 0 (36.2%). For each hospital, the HER2 IHC positive rate varied from 7.4 to 20.0%. The HER2 equivocal rate varied from 8.4 to 36.6%. The HER2 negative rate varied from 53.7 to 80.2%.

**Fig. 2:**
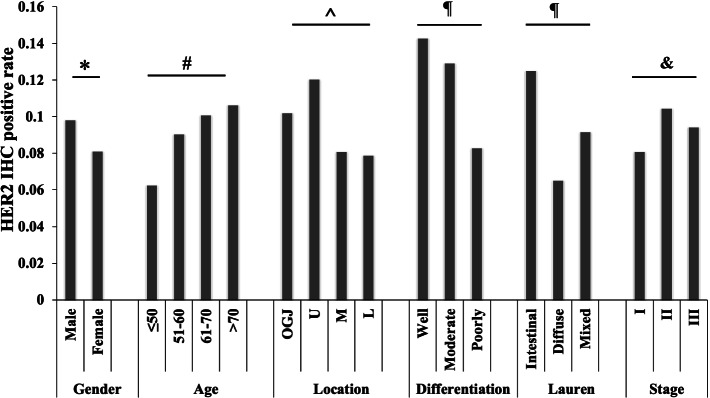
Comparison of HER2 IHC positive rate based on gender, age, tumor location, differentiation, Lauren classification, and stage. HER2 3+ rate was slightly higher in male than in female (*P = 0.113). The rate elevated with the increase of age (^**#**^*P* = 0.038). Higher positivity was found in GCs that located in the OGJ/U, and lower positive rate was shown in tumors that located in M/L (^*P* = 0.001). Low-grade tumors (well/moderate differentiation) and intestinal tumors were with higher HER2 positivity (^¶^*P* < 0.001). Stage did not affect the HER2 3+ rate (^&^*P* = 0.317). Abbreviations: Intestinal: Intestinal type; Diffuse: Diffuse type; Mixed: Mixed type; Indeterminate: Indeterminate type; OGJ: Oesophagogastric junction; U: The upper third of the stomach; M: The middle third of the stomach; L: The lower third of the stomach.

We next explored the correlations of HER2 IHC positive rate and clinicopathological parameters in total patients (Fig. 2). The HER2 positive rate was visually higher in male patients (9.8%) than in female patients (8.1%) without statistical significance (*P* = 0.113). The rate elevated with the increasing of patient age(≤50 6.3%; 51–60 9.0%; 61–70 10.1%; > 70 10.6%)(*P* = 0.038). With regard to tumor location, the HER2 positive rate of tumors in the oesophagogastric junction (OGJ)(10.2%) and the upper third of the stomach (12.0%) was much higher than that in the middle third (8.1%) and the lower third (7.9%) of the stomach (*P* = 0.001). Tumor differentiation and Lauren classification also affected the HER2 positivity. Higher HER2 positive rate was found in well (14.3%) and moderate (12.9%) differentiated tumors, while the poorly differentiated tumors showed the lowest positivity (8.3%) (*P* < 0.001). As to Lauren classification, intestinal type tumors were with the highest positive rate (12.5%), followed by mixed type (9.2%) and diffuse type (6.5%) respectively (*P* < 0.001). The positive rate did not show significant discrepancies among different stages (stage I 8.1%; stage II 10.4%; stage III 9.4%) (*P* = 0.317).

### Dual block assessment increased HER2 positive rate

**Fig. 3:**
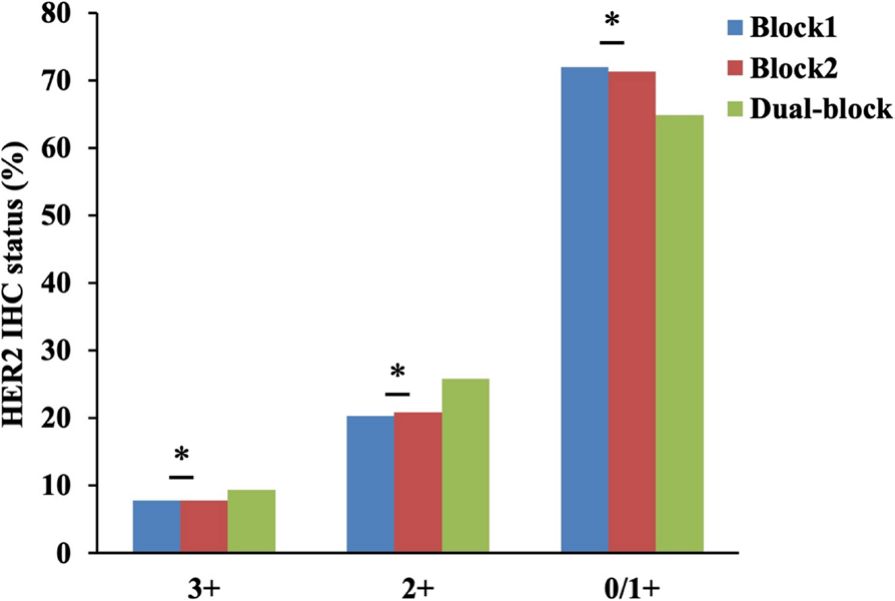
Comparison of HER2 status between single block assessment and dual block assessment in total patients. HER2 3+ rate of dual block assessment was significantly higher than that of both block 1 and block2 (*P < 0.001). HER2 2+ rate of dual block assessment was higher than that of both block 1 and block 2 in total patients (*P < 0.001). HER2 negative rate was lower in dual block assessment than in single block assessment (*P < 0.001)

The HER2 positive rate of dual block assessment and single block assessment was further compared in total patients (Table 2, Fig. 3) and each hospital (Supplementary Table 2, Supplementary Fig. 1A). In total patients, dual block assessment (9.4%) significantly increased the HER2 IHC positive rate compared with single block assessment (block 1, 7.8%; block 2, 7.8%) (*P* < 0.001 vs block1, 2). Subgroup analysis showed that in the 3 hospitals with more than 1000 patients, HER2 IHC positive (3+) rate was significant higher in dual block assessment than in single block assessment (Supplementary Table 2, Supplementary Fig. 1A). For the other 5 hospitals, the HER2 3+ rate was visually higher in dual block assessment without statistical significance (*P* > 0.05). However, after merging the patients of the 5 hospitals, HER 3+ rate of dual block assessment (9.9%) was significantly higher than both block 1 (8.3%) (*P* < 0.001) and block 2 (8.5%) (*P* < 0.001).

**Table 2:** Comparison of single block and dual block assessment in total patients

HER2 status	Block1	Block2	Dual-block	*P value* (Block1 vs Dual-block)	*P value* (Block2 vs Dual-block)
HER2 3+, n (%)	298 (7.8)	295 (7.8)	358 (9.4)	*P* < 0.001	*P* < 0.001
HER2 2+, n (%)	773 (20.3)	797 (20.9)	982 (25.8)	*P* < 0.001	*P* < 0.001
HER2 0/1+, n (%)	2735 (71.9)	2714 (71.3)	2466 (64.8)	*P* < 0.001	*P* < 0.001

### Dual block assessment increased HER2 equivocal rate

We next made the comparison of HER2 equivocal (2+) rate between dual block assessment and single block assessment in total patients (Table. 2, Fig. 3) and each hospital (Supplementary Table 2, Supplementary Fig. 1B). Similar to HER2 positive rate, HER2 equivocal rate was increased by dual block assessment in total patients. The rate increased from 20.3% (block 1) or 20.9% (block 2) to 25.8% (*P* < 0.001 vs block1 or block2). The separate evaluation of each hospital indicated that in 5 of the 8 hospitals HER2 equivocal rate was effectively elevated (*P* < 0.05). In 2 hospitals, dual block assessment demonstrated statistically higher HER2 equivocal rate compared with either block1 or block2 and visually higher the rate compared with the other group. In the remaining one hospital, dual block assessment only exhibited visual increase of HER2 2+ rate without statistical significance (*P* > 0.05).

### Dual block assessment decreased HER2 negative rate

We then evaluated how dual block assessment affected HER2 negative (0/1+) rate in total patients (Table 2, Fig. 3) and each hospital (Supplementary Table 2, Supplementary Fig. 1C). In total patients, the HER2 negative rate was significantly reduced (*P* < 0.001 vs block1 or block 2). Further analysis showed that the rate exhibited similar decreasing changes in dual block assessment compared with the two single block groups in 7 of the 8 hospitals (*P* < 0.05). In the remaining hospital, HER2 negative rate of single block assessment was visually higher than that of dual block assessment, but statistical difference was only reached in the comparison between block 2 and dual block assessment (*P* = 0.25 vs block1, *P* = 0.002 vs block 2).

### HER2 heterogeneity

HER2 heterogeneity was also evaluated in total patients (Table.3). HER2 homogeneous expression (concordant HER2 results between the two blocks) was found in 2823 cases (74.2%), and HER2 heterogeneity (discordant HER2 results between the two blocks) was shown in 983 cases (25.8%) (Table.4). Among them, with regard to the HER2 results of the two blocks, 411 cases showed 0 and 1+ (41.8% of the heterogeneous cases); 129 cases showed 0 and 2+ (13.1% of the heterogeneous cases); 45 cases showed 0 and 3+ (4.6% of the heterogeneous cases); 326 cases showed 1+ and 2+ (33.2% of the heterogeneous cases); 27 cases showed 1+ and 3+ (2.7% of the heterogeneous cases); 45 cases showed 2+ and 3+ (4.6% of the heterogeneous cases).

**Table 3:** Comparison of HER2 results between the two blocks

		Block 2, n (%)				Total, n (%)
		0	1+	2+	3+	
Block 1, n (%)	0	1379 (36.2)	241 (6.3)	67 (1.8)	22 (0.6)	1709 (44.9)
	1+	170 (4.5)	671 (17.6)	173 (4.5)	12 (0.3)	1026 (27.0)
	2+	62 (1.6)	153 (4.0)	535 (14.1)	23 (0.6)	773 (20.3)
	3+	23 (0.6)	15 (0.4)	22 (0.6)	238 (6.3)	298 (7.8)
Total, n (%)		1634 (42.9)	1080 (28.4)	797 (20.9)	295 (7.8)	3806 (100)

**Table 4:** Concordance and discordance of HER2 expression in dual block assessment in total patients

	Case number, n (%)
Concordant	2823 (74.2)
Discordant	983 (25.8)
0 vs 1+	411 (41.8)
0 vs 2+	129 (13.1)
0 vs 3+	45 (4.6)
1 vs 2+	326 (33.2)
1 vs 3+	27 (2.7)
2 vs 3+	45 (4.6)

## Discussion

This is a prospective multi institutional study based on real world data. It is also a follow-up study of our previous single center studies. To our knowledge, this is the first multi-center study to explore the value of multiple-block HER2 assessment in GC. The results showed that HER2 positive rate and equivocal rate was increased by dual block assessment and meanwhile the HER2 negative rate was decreased. These findings were confirmed in total patients as well as in each hospital. Therefore, dual block assessment could not only increase HER2 IHC positive rate directly, but also may increase the number of HER2 equivocal cases which could be subjected to FISH analysis.

These findings were consistent with our former single center studies [21, 22]. As a large-scale multi-center clinical trial, a total of 3806 patients were enrolled from 8 hospitals, which provided a large enough sample size to draw the conclusions. The consistency of the impact of dual block assessment on HER2 analysis across institutions further proved the generalizability of this methodology. In several institutions, dual block assessment led to visually rather than statistically different HER2 results. This was most likely duo to relatively small sample size, and statistical significance was reached after merging these institutions together.

Data of this study was collected from real-world patients. To verify its reliability, we analyzed the basic characteristics of HER2 in this study. The results showed that HER2 status of this study was consistent with former studies. Briefly, HER2 IHC positive rate was higher in male patients, intestinal type tumors, low-grade tumors, and tumors locating in the GEJ or the upper third of the stomach [26–28]. The rate was also associated with age and was lower in younger patients, which was indicated in several studies [29, 30]. These findings help confirm the reliability of our data and strengthen the conclusions.

Therefore, dual block assessment is an effective and efficient method to increase HER2 positive rate. However, increasing HER2 positive rate is not the ultimate goal of HER2 analysis. To get eligible patients for the molecular therapy is major purpose. If the extra gained patients selected by dual block assessment do not benefit from the treatments, simply increasing the positive rate is of limited clinical significance. About the impact of HER2 heterogeneity on trastuzumab efficacy, there have been several studies with controversial findings. Some studies indicated that HER2 heterogeneity was a negative predictor to anti-HER2 targeted therapy [31, 32].However, some other studies showed different conclusions. Van Cutsem et al. analyzed the data from ToGA, and found that staining intensity variability of HER2 did not affect the overall benefit of trastuzumab [33]. Another study showed that HER2 heterogeneity alone did not affect the efficacy of trastuzumab treatment [34]. A recent study from our team indicated that late stage GC patients with extra gained HER2 positivity by dual block assessment may not show compromised efficacy to trastuzumab treatment [35]. These studies supported that heterogeneity may not affect trastuzumab efficacy, and patients with newly diagnosed HER2 positivity may acquire similar benefit from anti-HER2 targeted therapy, therefore, it is reasonable to regard these patients as eligible candidates for the targeted therapy.

Not only in GC, even in breast cancer (BC), there were also controversies in the predictability of HER2 heterogeneity. Although most studies supported that BC with homogeneously HER2 positivity benefited more from trastuzumab treatment [13], predictive relationship between the genetic heterogeneity and treatment response was not found in early stage breast cancers in the adjuvant setting [36]. In addition, the cut off to define HER2 IHC positive (3+) in BC was adjusted from 30 to 10% of the invasive tumor cells [37]. This change indicated that heterogeneously positive BC could benefit from the targeted treatment.

Therefore, clinically, it is reasonable to regard HER2 heterogeneously positive GC patients as the potential eligible candidates for the molecular targeted therapy. It might be imprudent to deprive these cases with heterogeneous positivity of the opportunity to get the targeted treatment based on current data from relatively small-scale perspective studies. Considering for GC, trastuzumab was the only confirmed targeted regimen to show benefit currently [38–40], to acquire more eligible patients for the treatment is of great clinical significance.

Dual block assessment provided a feasible and simple way to identify more HER2 positive patients by identifying more heterogeneously positive cases. As shown in this study, using IHC alone, the methodology could increase the positive rate by 20.1% (from 7.8 to 9.4%). With the identification of more HER2 heterogeneously positive cases and the following targeted therapy, the impact of HER2 heterogeneity on the treatment efficacy can be further explored and large-scale prospective studies are expected.

In addition, dual block assessment is a cost-effective method to increase HER2 positivity. As shown in our former studies, by putting the two blocks on one slide, IHC tests for both blocks can be achieved simultaneously without consuming extra reagents [21, 22]. Dual-block assessment is therefore an efficient and practical method to provide more accurate HER2 results without much additional effort or expense.

HER2 testing in GC is an evolving and constantly improving paradigm. Recently, the number of biopsy specimens required to get reliable HER2 results has been discussed in the current guidelines for HER2 assessment [11, 23, 25, 41]. However, little was discussed regarding to the ideal number of tumor-containing blocks to be tested in resected specimens of GC. After the first proposal of the concept of dual block assessment, it has been discussed as a feasible option to deal with HER2 heterogeneity in several guidelines for HER2 assessment in GC [12, 42, 43]. It had not been accepted as a formal consensus in HER2 analysis worldwide yet. The current study as well as our previous single-center studies [21, 22] provided solid evidence that dual block assessment can be served as a new reliable strategies to deal with the adverse impacts of HER2 heterogeneity in GC which may potentially perfect future guidelines.

In this study, IHC staining was used in the HER2 assessment for its efficiency and high concordance to FISH [44]. In clinical practice, HER2 2+ cases should be subjected to FISH assessment. As shown in this study, dual block assessment also increased HER2 2+ rate, indicating that more candidates would undertake FISH analysis which may further increase HER2 amplification rate. The HER2 amplification rate in 2+ patients varied widely from less than 10% to 30–50% in previous studies, partly due to the differences in histological subtypes and antibodies, partly due to subjective interpretation of IHC results [12, 22, 27, 45–47]. In Chinese patients, several publications indicated that the amplification rate of 2+ cases was relatively lower (less than 10 to 20%) [20, 22, 27, 48]. In our previous retrospective study, a few newly diagnosed HER2 2+/FISH+ patients by dual block assessment did not show compromised efficacy to anti-HER2 targeted therapy [35]. Therefore, besides increasing HER2 3+ rate, increasing HER2 equivocal (2+) rate is another clinical value of dual block assessment.

In conclusion, the multi institutional clinical trial proved that dual block assessment increases HER2 IHC positive rate and equivocal rate and meanwhile decreases the HER2 negative rate. Therefore, more eligible patients will be identified for the molecular target therapy. It is a simple, effective and practical way to minimize the impacts of HER2 heterogeneity by simply adding one tumor-containing block in HER2 assessment. We recommended dual block assessment routinely performed in the resected specimens in GC to reduce false negative rate.

## Supplementary Information

**Additional file 1: Supplementary Figure 1.** Comparison of HER2 status between single block assessment and dual block assessment in each hospital. A. The comparison of HER2 3+ rate. In 2 hospitals including ZH and HCH, HER2 3+ rate of dual block assessment was significantly higher than that of both block 1 and block2 (*P < 0.001). In SRRSH, HER2 3+ rate of dual block assessment was statistically higher than that of block2 (^**#**^*P* < 0.05) but only visually higher than that of block 1 (^*P* > 0.05). For the other 5 hospitals, HER2 3+ rate of dual block assessment was higher than single block assessment without statistical significance (^*P* > 0.05). B. The comparison of HER2 2+ rate. HER2 2+ rate of dual block assessment was higher than that of both block 1 and block 2 in 4 hospitals (ZH, HCH, SH and FAHZU) (*P < 0.001, ^**#**^*P* < 0.05). The rate of dual block was statistically higher either block 1 or block 2 in SRRSH and ZCH (^**#**^*P* < 0.05, ^*P* > 0.05). In FAHAMU and SAHZU, HER2 2+ rate was visually higher in dual block assessment than in single block assessment without statistical significance (^*P* > 0.05). C. The comparison of HER2 0/1+ rate. In 7 hospitals (ZH, HCH, SRRSH, XH, FAHAMU, FAHZU, and SAHZU), HER2 negative rate was lower in dual block assessment than in single block assessment (*P < 0.001, ^**#**^*P* < 0.05). In ZCH, statistical difference was only shown in the comparison between dual block and block 2 (^**#**^*P* < 0.05, ^*P* > 0.05). Abbreviations: ZH: Zhongshan Hospital, Fudan University; HCH: Henan Cancer Hospital; SRRSH: Sir Run Run Shaw Hospital; XH: Xijing Hospital; FAHAMU: The First Affiliated Hospital of Anhui Medical University; FAHZU: The First Affiliated Hospital of Zhejiang University; ZCH: Zhejiang Cancer Hospital; SAHZU: The Second Affiliated Hospital of Zhejiang University.

**Additional file 2: Supplementary Table 1.** Patient characteristics of each hospital.

**Additional file 3: Supplementary Table 2.** Comparisons of single block and dual block assessment in each hospital.

## Data Availability

All data are included in the author’s manuscript.

## Abbreviations

HER2: Human epidermal growth receptor 2
GC: Gastric cancer
BC: Breast cancer
IHC: Immunohistochemistry
FISH: Fluorescence in situ hybridization
SISH: Silver in situ hybridization
