# Supplementary figures and images for: Dual block HER2 assessment increased HER2 immunohistochemistry positive rate in resected specimens of gastric cancer: a prospective multicenter clinical trial from China
Source: Diagn Pathol. 2022 Jun 28;17:54. doi: 10.1186/s13000-022-01230-7 (PMC9238183; doi:10.1186/s13000-022-01230-7)

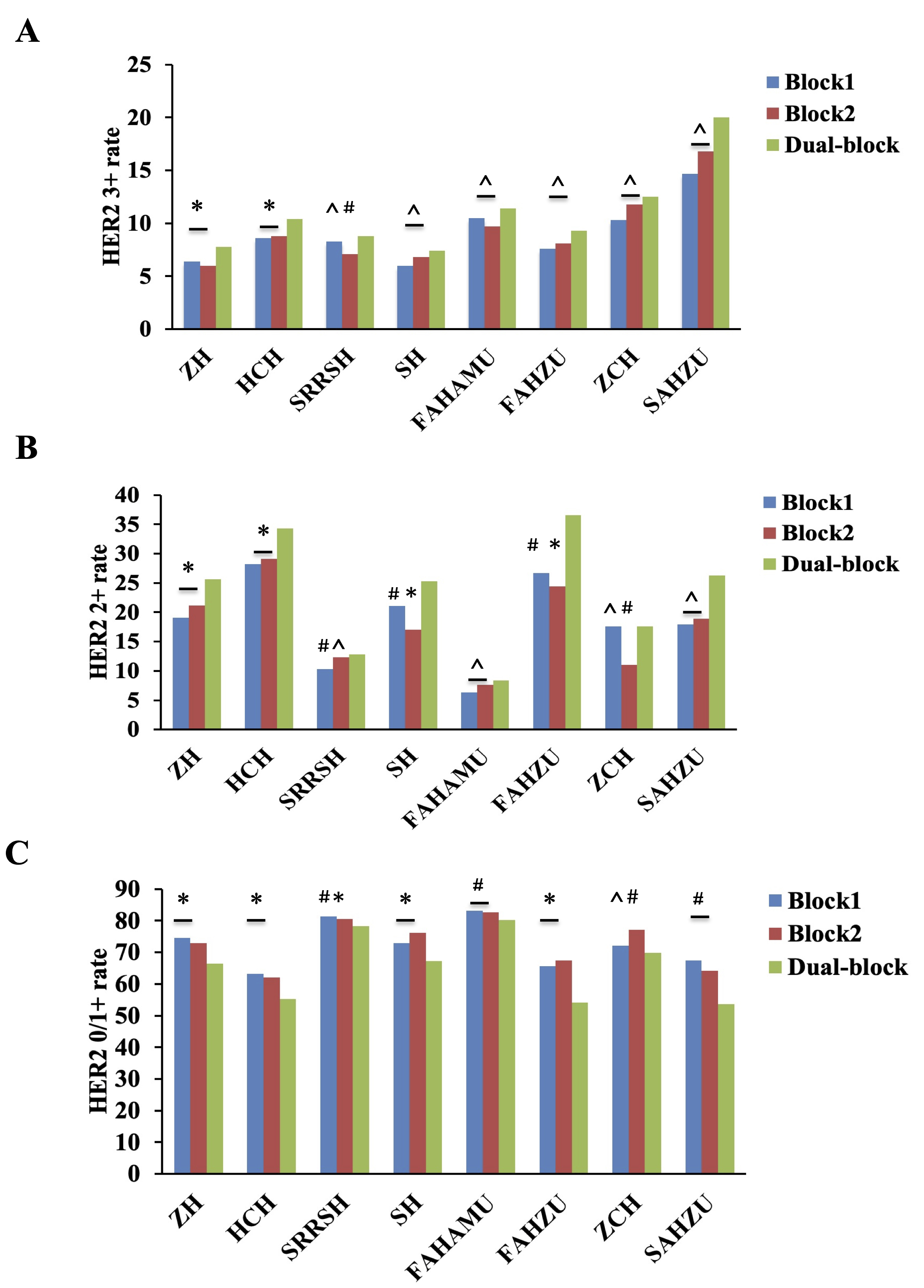

Supplement: Supplementary file 1 — Additional file 1: Supplementary Figure 1. Comparison of HER2 status between single block assessment and dual block assessment in each hospital. A. The comparison of HER2 3+ rate. In 2 hospitals including ZH and HCH, HER2 3+ rate of dual block assessment was significantly higher than that of both block 1 and block2 (*P < 0.001). In SRRSH, HER2 3+ rate of dual block assessment was statistically higher than that of block2 (#P < 0.05) but only visually higher than that of block 1 (^P > 0.05). For the other 5 hospitals, HER2 3+ rate of dual block assessment was higher than single block assessment without statistical significance (^P > 0.05). B. The comparison of HER2 2+ rate. HER2 2+ rate of dual block assessment was higher than that of both block 1 and block 2 in 4 hospitals (ZH, HCH, SH and FAHZU) (*P < 0.001, #P < 0.05). The rate of dual block was statistically higher either block 1 or block 2 in SRRSH and ZCH (#P < 0.05, ^P > 0.05). In FAHAMU and SAHZU, HER2 2+ rate was visually higher in dual block assessment than in single block assessment without statistical significance (^P > 0.05). C. The comparison of HER2 0/1+ rate. In 7 hospitals (ZH, HCH, SRRSH, XH, FAHAMU, FAHZU, and SAHZU), HER2 negative rate was lower in dual block assessment than in single block assessment (*P < 0.001, #P < 0.05). In ZCH, statistical difference was only shown in the comparison between dual block and block 2 (#P < 0.05, ^P > 0.05). Abbreviations: ZH: Zhongshan Hospital, Fudan University; HCH: Henan Cancer Hospital; SRRSH: Sir Run Run Shaw Hospital; XH: Xijing Hospital; FAHAMU: The First Affiliated Hospital of Anhui Medical University; FAHZU: The First Affiliated Hospital of Zhejiang University; ZCH: Zhejiang Cancer Hospital; SAHZU: The Second Affiliated Hospital of Zhejiang University. [file 13000_2022_1230_MOESM1_ESM.jpg]
