# Supplementary material for: Dual block HER2 assessment increased HER2 immunohistochemistry positive rate in resected specimens of gastric cancer: a prospective multicenter clinical trial from China
Source: Diagn Pathol. 2022 Jun 28;17:54. doi: 10.1186/s13000-022-01230-7 (PMC9238183; doi:10.1186/s13000-022-01230-7)
Supplement: Supplementary file 2 — Additional file 2: Supplementary Table 1. Patient characteristics of each hospital. [file 13000_2022_1230_MOESM2_ESM.docx]

Supplementary table.1 Patient characteristics of each hospital

|  | Zhongshan Hospital | Henan Cancer Hospital | Sir Run Run Shaw Hospital | Xijing Hospital | The First Affiliated Hospital of Anhui Medical University | The First Affiliated Hospital of Zhejiang University | Zhejiang Cancer Hospital | The Second Affiliated Hospital of Zhejiang University |
| --- | --- | --- | --- | --- | --- | --- | --- | --- |
| Total, n (%) | 1389 (36.5) | 1044 (27.4) | 397 (10.4) | 336 (8.8) | 237 (6.2) | 172 (4.5) | 136 (3.6) | 95 (2.5) |
| Gender, n (%) |  |  |  |  |  |  |  |  |
| Male | 1014 (73.0) | 796 76.2) | 281 (70.8) | 268 (79.8) | 173 (73.0) | 123 (71.5) | 99 (72.8) | 66 (69.5) |
| Female | 375 (27.0) | 248 (23.8) | 116 (29.2) | 68 (20.2) | 64 (27.0) | 49 (28.5) | 37 (27.2) | 29 (30.5) |
| Differentiation, n (%) |  |  |  |  |  |  |  |  |
| Well | 27 (1.9) | 34 (3.3) | 26 (6.5) | 14 (4.2) | 2 (0.8) | 0 (0.0) | 0 (0.0) | 9 (9.5) |
| Moderate | 243 (17.5) | 242 (23.2) | 76 (19.1) | 82 (24.4) | 46 (19.4) | 17 (9.9) | 30 (22.1) | 15 (15.8) |
| Poorly | 1119 (80.6) | 768 (73.6) | 295 (74.3) | 240 (71.4) | 189 (79.7) | 155 (90.1) | 106 (77.9) | 71 (74.7) |
| Lauren, n (%) |  |  |  |  |  |  |  |  |
| Intestinal | 577 (41.5) | 345 (33.0) | 103 (25.9) | 114 (33.9) | 39 (16.5) | 66 (38.4) | 70 (51.5) | 36 (37.9) |
| Diffuse | 313 (22.5) | 391 (37.5) | 144 (36.3) | 86 (25.6) | 98 (41.4) | 70 (40.7) | 63 (46.3) | 32 (33.7) |
| Mixed | 455 (32.8) | 308 (29.5) | 139 (35.0) | 13 (36.6) | 100 (42.2) | 35 (20.3) | 3 (2.2) | 26 (27.4) |
| Indeterminate | 44 (3.2) | 0 (0.0) | 0 (0.0) | 13 (3.9) | 0 (0.0) | 1 (0.6) | 0 (0.0) | 1 (1.1) |
| Location, n (%) |  |  |  |  |  |  |  |  |
| OGJ | 26 (1.91) | 142 (13.6) | 3 (0.8) | 0 (0.0) | 10 (4.2) | 2 (1.2) | 1 (0.7) | 2 (2.1) |
| U | 461 (33.2) | 461 (44.2) | 59 (14.9) | 112 (33.3) | 99 (41.8) | 26 (15.1) | 34 (25.0) | 10 (10.5) |
| M | 129 (9.3) | 160 (15.3) | 65 (16.4) | 56 (16.7) | 52 (21.9) | 36 (20.9) | 15 (11.0) | 31 (32.6) |
| L | 772 (55.6) | 281 (26.9) | 270 (68.0) | 168 (50.0) | 65 (27.4) | 108 (62.8) | 86 (63.2) | 52 (54.7) |
| Others | 1 (0.1) | 0 (0.0) | 0 (0.0) | 0 (0.0) | 11 (4.6) | 0 (0.0) | 0 (0.0) | 0 (0.0) |
| Stage, n (%) |  |  |  |  |  |  |  |  |
| IA | 298 (21.5) | 84 (8.0) | 64 (16.1) | 64 (19.0) | 9 (3.8) | 30 (17.4) | 10 (7.4) | 14 (14.7) |
| IB | 150 (10.8) | 91 (8.7) | 39 (9.8) | 27 (8.0) | 21 (8.9) | 16 (9.3) | 13 (9.6) | 9 (9.5) |
| IIA | 176 (12.7) | 55 (5.3) | 29 (7.3) | 51 (15.2) | 17 (7.2) | 27 (15.7) | 13 (9.6) | 22 (23.2) |
| IIB | 187 (13.5) | 219 (21.0) | 47 (11.8) | 42 (12.5) | 39 (16.5) | 30 (17.4) | 19 (14.0) | 13 (13.7) |
| IIIA | 184 (13.2) | 362 (34.7) | 120 (30.2) | 45 (13.4) | 39 (16.5) | 16 (9.3) | 9 (6.6) | 13 (13.7) |
| IIIB | 175 (12.6) | 180 (17.2) | 71 (17.9) | 45 (13.4) | 45 (19.0) | 34 (19.8) | 25 (18.4) | 15 (15.8) |
| IIIC | 219 (15.8) | 52 (5.0) | 27 (6.8) | 62 (18.5) | 67 (28.3) | 17 (9.9) | 46 (33.8) | 7 (7.4) |
| IV | 0 (0.0) | 1 (0.1) | 0 (0.0) | 0 (0.0) | 0 (0.0) | 2 (1.2) | 1(0.7) | 2 (2.1) |
| HER2, n (%) |  |  |  |  |  |  |  |  |
| 3+ | 109 (7.8) | 109 (10.4) | 35 (8.8) | 25 (7.4) | 27 (11.4) | 17 (9.9) | 17 (12.5) | 19 (20.0) |
| 2+ | 356 (25.6) | 358 (34.3) | 51 (12.8) | 85 (25.3) | 20 (8.4) | 63 (36.6) | 24 (17.6) | 25 (26.3) |
| 1+ | 503 (36.2) | 221 (21.2) | 100 (25.2) | 94 (28.0) | 48 (20.3) | 50 (29.1) | 38 (27.9) | 37 (38.9) |
| 0 | 421 (30.3) | 356 (34.1) | 211 (53.1) | 132 (39.3) | 142 (59.9) | 43 (25.0) | 57 (41.9) | 14 (14.7) |

Abbreviations: Intestinal: Intestinal type; Diffuse: Diffuse type; Mixed: Mixed type; Indeterminate: Indeterminate type; OGJ: Oesophagogastric junction; U: The upper third of the stomach; M: The middle third of the stomach; L: The lower third of the stomach
