# Supplementary material for: Dual block HER2 assessment increased HER2 immunohistochemistry positive rate in resected specimens of gastric cancer: a prospective multicenter clinical trial from China
Source: Diagn Pathol. 2022 Jun 28;17:54. doi: 10.1186/s13000-022-01230-7 (PMC9238183; doi:10.1186/s13000-022-01230-7)
Supplement: Supplementary file 3 — Additional file 3: Supplementary Table 2. Comparisons of single block and dual block assessment in each hospital. [file 13000_2022_1230_MOESM3_ESM.docx]

Supplementary table.2 Comparisons of single block and dual block assessment in each hospital.

|  | Block1 | Block2 | Dual-block | *P value* (Block1 *vs* Dual-block) | *P value* (Block2 *vs* Dual-block) |
| --- | --- | --- | --- | --- | --- |
| HER2 3+, n (%) |  |  |  |  |  |
| Zhongshan Hospital | 89 (6.4) | 83 (6.0) | 109 (7.8) | ***P*<0.001** | ***P*<0.001** |
| Henan Cancer Hospital | 90 (8.6) | 92 (8.8) | 109 (10.4) | ***P*<0.001** | ***P*<0.001** |
| Sir Run Run Shaw Hospital | 33 (8.3) | 28 (7.1) | 35 (8.8) | *P*=0.5 | ***P*=0.016** |
| Xijing Hospital | 20 (6.0) | 23 (6.8) | 25 (7.4) | *P*=0.063 | *P*=0.5 |
| The First Affiliated Hospital of Anhui Medical University | 25 (10.5) | 23 (9.7) | 27 (11.4) | *P*=0.5 | *P*=0.125 |
| The first Affiliated Hospital of Zhejiang University | 13 (7.6) | 14 (8.1) | 17 (9.9) | *P*=0.125 | *P*=0.25 |
| Zhejiang Cancer Hospital | 14 (10.3) | 16 (11.8) | 17 (12.5) | *P*=0.25 | *P*=1.0 |
| The Second Affiliated Hospital of Zhejiang University | 14 (14.7) | 16 (16.8) | 19 (20.0) | *P*=0.063 | *P*=0.25 |
| HER2 2+, n (%) |  |  |  |  |  |
| Zhongshan Hospital | 265 (19.1) | 294 (21.2) | 356 (25.6) | ***P*<0.001** | ***P*<0.001** |
| Henan Cancer Hospital | 294 (28.2) | 304 (29.1) | 358 (34.3) | ***P*<0.001** | ***P*<0.001** |
| Sir Run Run Shaw Hospital | 41 (10.3) | 49 (12.3) | 51 (12.8) | ***P*=0.002** | *P*=0.727 |
| Xijing Hospital | 71 (21.1) | 57 (17.0) | 85 (25.3) | ***P*=0.003** | ***P*<0.001** |
| The First Affiliated Hospital of Anhui Medical University | 15 (6.3) | 18 (7.6) | 20 (8.4) | *P*=0.125 | *P*=0.5 |
| The first Affiliated Hospital of Zhejiang University | 46 (26.7) | 42 (24.4) | 63 (36.6) | ***P*=0.001** | ***P*<0.001** |
| Zhejiang Cancer Hospital | 24 (17.6) | 15 (11.0) | 24 (17.6) | *P*=1.0 | ***P*=0.004** |
| The Second Affiliated Hospital of Zhejiang University | 17 (17.9) | 18 (18.9) | 25 (26.3) | *P*=0.057 | *P*=0.065 |
| HER2 0/1+, n (%) |  |  |  |  |  |
| Zhongshan Hospital | 1035 (74.5) | 1012 (72.9) | 924 (66.5) | ***P*<0.001** | ***P*<0.001** |
| Henan Cancer Hospital | 660 (63.2) | 648 (62.1) | 577 (55.3) | ***P*<0.001** | ***P*<0.001** |
| Sir Run Run Shaw Hospital | 323 (81.4) | 320 (80.6) | 311 (78.3) | ***P*<0.001** | ***P*=0.004** |
| Xijing Hospital | 245 (72.9) | 256 (76.2) | 226 (67.3) | ***P*<0.001** | ***P*<0.001** |
| The First Affiliated Hospital of Anhui Medical University | 197 (83.1) | 196 (82.7) | 190 (80.2) | ***P*=0.016** | ***P*=0.031** |
| The first Affiliated Hospital of Zhejiang University | 113 (65.7) | 116 (67.4) | 92 (53.5) | ***P*<0.001** | ***P*<0.001** |
| Zhejiang Cancer Hospital | 98 (72.1) | 105 (77.2) | 95 (69.9) | *P*=0.25 | ***P*=0.002** |
| The Second Affiliated Hospital of Zhejiang University | 64 (67.4) | 61 (64.2) | 51 (53.7) | ***P*=0.001** | ***P*=0.006** |
